# Rhododendron poisoning in alpacas (*Vicugna pacos*) in Northern Germany

**DOI:** 10.1007/s11259-024-10334-y

**Published:** 2024-03-14

**Authors:** Johannes Schregel, Isabel Zdora, Ingo Gerhauser, Teresa Maria Punsmann, Sabine Aboling, Martin Ganter, Matthias Gerhard Wagener

**Affiliations:** 1 Clinic for Swine and Small Ruminants, Forensic Medicine and Ambulatory Service, University of Veterinary Medicine Hannover, Foundation, Hanover, Germany; 2 Department of Pathology, University of Veterinary Medicine Hannover, Foundation, Hanover, Germany; 3 Institute for Institute for Animal Nutrition, University of Veterinary Medicine Hannover, Foundation, Hanover, Germany

**Keywords:** Alpaca, Poisoning, rhododendron, Clinical chemistry, Pathology

## Abstract

Poisoning is often suspected to be the origin of disease in South American camelids (SACs) by owners, but only in a few cases this assumption can be confirmed. In small ruminants, rhododendron poisoning is a common emergency for livestock veterinarians. However, this condition has rarely been reported in SACs so far. This paper provides information regarding clinical findings, hematology, clinical chemistry, and treatment of four alpacas after presumed intake of rhododendron leaves including pathological findings of one of the animals. Rhododendron leaves contain grayanatoxins that lead to hyperpolarization of excitable cells. Clinical signs that were observed in the presented alpacas comprised: salivation, dehydration, decreased motility of compartment 1, uncoordinated regurgitation, and cardiac arrhythmia. Clinical chemistry revealed that rhododendron poisoning was associated with metabolic acidosis and azotaemia, hyponatremia and hyperkalemia. Most striking macroscopic and histopathological findings included gastric ulceration, and renal infarcts along with inflammatory changes. Leaves of *Rhododendron* spp. were identified in the forestomach content of this animal. Affected animals were treated symptomatically as there is no specific antidote in rhododendron poisoning. This included parenteral rehydration, treatment of metabolic acidosis (infusion of sodium bicarbonate solution), and oral administration of activated charcoal to bind potential toxins. In addition, antibiotic treatment might be necessary to prevent aspiration pneumonia in case of uncoordinated regurgitation. Of the four animals, the worst affected alpaca was euthanized, one had minimal signs and two responded to supportive care and recovered. In conclusion, rhododendron poisoning might be fatal for alpacas in individual cases and therefore rhododendron bushes should not be placed in the habitat of SACs.

## Introduction

When South American camelids (SACs) are presented to the clinic, owners often suspect poisoning in the anamnesis. Nevertheless, poisoning is extremely rare, which has been demonstrated by a study conducted in the United Kingdom based on laboratory data obtained from SACs (Twomey et al. 2014). Only 15 cases (0.29%) of 5154 samples tested in 11 years were diagnosed as “poisoning”. Out of these 15 cases, seven were due to copper poisoning and eight cases due to plant poisoning (0.15%) with one each due to cherry laurel (*Prunus laurocerasus*), cotoneaster (*Cotoneaster* spp.), ryegrass (*Lolium* spp.) (ryegrass staggers), yew (*Taccus bacta*), rhododendron (*Rhododendron* spp.). In three cases the plant species could not be identified. There are only few isolated case reports of intoxications in SACs. These mostly resulted from ornamental plants such as rhododendron (*Rhododendron* spp.) (Crawford 1999), red maple (*Acer rubrum*) (Dewitt et al. 2004), oleander (*Nerium oleander*) (Kozikowski et al. 2009), cotoneaster (*Cotoneaster* spp.) (Grüss and Priymenko 2009), South African Bornholm daisy (*Osteospermum ecklonis*) (Mc Kenzie et al. 2009), *Rumex spp.* (Mitsui et al. 2007) or Chilean hammerbush (*Cestrum parqui*) (McNiven R et al. 1993). Less commonly, poisoning by cultivated species such as oats (*Avena sativa*) (Mc Kenzie et al. 2009) or black oil sunflower seeds (*Helianthus annuus L.*) (McKenzie et al. 2021) have been reported. In addition, cases of poisoning in alpacas have been associated with fungal toxins. Susceptibility of SACs to poisoning with *Neotyphodium lolii*, an endophyte living in symbiosis with ryegrasses (*Lolium* spp.), has also been described (Sampaio et al. 2008). Additionally, the terpenoid cantharidin originating from beetles such as *Epicauta occidentalis* has been associated with poisoning in SACs (Simpson et al. 2013). Poisoning of SACs can also occur due to alimentary or iatrogenic oversupply of vitamin D_3_ (Gerspach et al. 2010; Wagener et al. 2021) or propylene glycol (Ivany and Anderson 2001). There is a lack of information regarding the selection of feed in SACs in humid and oceanic habitats like in Northwestern Germany. Alpacas are classic opportunistic mixed grazers, which feed on grasses, herbs, and woody plants in their natural habitat, but are restricted to woody plants during the dry season (Castellaro et al. 2008). However, the feeding behavior of SACs, differs from that of small ruminants (Stölzl et al. 2015, 2017). The first evidence of toxicity of rhododendron to humans has been known since ancient times and was already described by the Greek philosopher Xenophon (Jansen et al. 2012). However, there have been very few data on rhododendron poisoning in alpacas. Crawford (1999) reported on a herd of alpacas kept on a pasture with rhododendron bushes (Crawford 1999). Within 24 h, salivation, ataxia, and colic were observed and rhododendron leaves were found in the stomach contents of two animals that died four days later. According to Lakritz, rhododendron poisoning in alpacas is associated with uncoordinated regurgitation, colic, paresis, anorexia as well as convulsions (Lakritz 2022).

The andromedotoxin (or grayanotoxin or acetylandromedol) contained in rhododendron leaves (Popescu and Kopp 2013) binds to voltage-dependent sodium channels and causes permanent hyperpolarization of excitable cells such as neurons, myocytes, and cardiomyocytes (Jansen et al. 2012). This hyperpolarization inhibits the transmission of excitation in the heart as well as paralysis of striated muscles, which ultimately affects respiration (Puschner 2003). Rhododendron poisoning in sheep and goats is more common than in alpacas. Symptoms in small ruminants include uncoordinated regurgitation, salivation, dyspnea, tachycardia, arrhythmia, and ataxia (Hosie et al. 1986; Eo and Kwon 2009). In addition, symptoms of poisoning after rhododendron ingestion have also been described in other species such as donkeys (Thiemann 1991), kangaroos (Hough 1997), dogs (Frape and Ward 1993), and tortoises (Brown 2006) .

To our knowledge, this is the first detailed description of rhododendron intoxication in SACs. In the following, clinical, laboratory diagnostic as well as pathological findings of rhododendron poisoning in alpacas from Northern Germany will be presented.

## Case description

### Medical history

Over a two-year period, four alpacas were hospitalized with suspected rhododendron poisoning in our clinic.

Three of these animals came from the same farm (farm 1) and were kept on different pastures. In addition to pasture, hay and a special concentrate mixture for alpacas were available to the animals. The animals were all regularly vaccinated against clostridia (Covexin 10, Zoetis Deutschland GmbH, Berlin, Germany) and regularly dewormed. Animals from farm 1 are the following:

Alpaca 1: Huacaya: female; six years old; 76.5 kg body weight (BW);

Alpaca 2: Huacaya: female; four years old; 73 kg BW;

Alpaca 3: Huacaya: female; eight years old; 51.5 kg BW.

These animals had been grazing on a pasture with access to rhododendron bushes for seven days prior to admission to the clinic on June 9,2020. In the afternoon on the day of admission to the clinic, two of the animals started showing abnormal behavior. According to the animal owner, alpaca 1 was apathetic, shook its head, and rolled over its back several times. Furthermore, the owner observed foaming discharge from the mouth, bruxism, coughing, and retching. Alpaca 2 showed intermittent bruxism and foaming discharge from the mouth. No abnormalities were observed in alpaca 3. The owner also reported bite marks on rhododendron bushes. Initial therapy included oral administration of charcoal and application of a spasmolytic (butylscopolaminiumbromid) combined with a non-steroidal anti-inflammatory drug (NSAID, metamizole-sodium) and was performed by the local veterinarian. Alpaca 3 was additionally treated with oral administration of vegetable oil and caraway by the local veterinarian. Since the condition of both alpaca 1 and 2 deteriorated, they were presented to the clinic late that evening.

The fourth animal was a 1.5-year-old male alpaca (alpaca 4: Huacaya: two years old, 60 kg BW) from another hobby farm (farm 2). The flock consisted of three animals that had only been kept on the farm for four months. These animals were kept on rotating pastures during the day. In addition to grass, hay and a special concentrate mixture for alpacas were also available to the animals at night. The animals in the herd were all regularly vaccinated against clostridia (Covexin 10, Zoetis Deutschland GmbH) and regularly dewormed. This animal was transported to the clinic in the afternoon of November 30, 2022. The owner reported intense salivation, gagging, and foaming discharge at the mouth and the animal was recumbent in a sternal position.

### Clinical findings

**Fig. 1:**
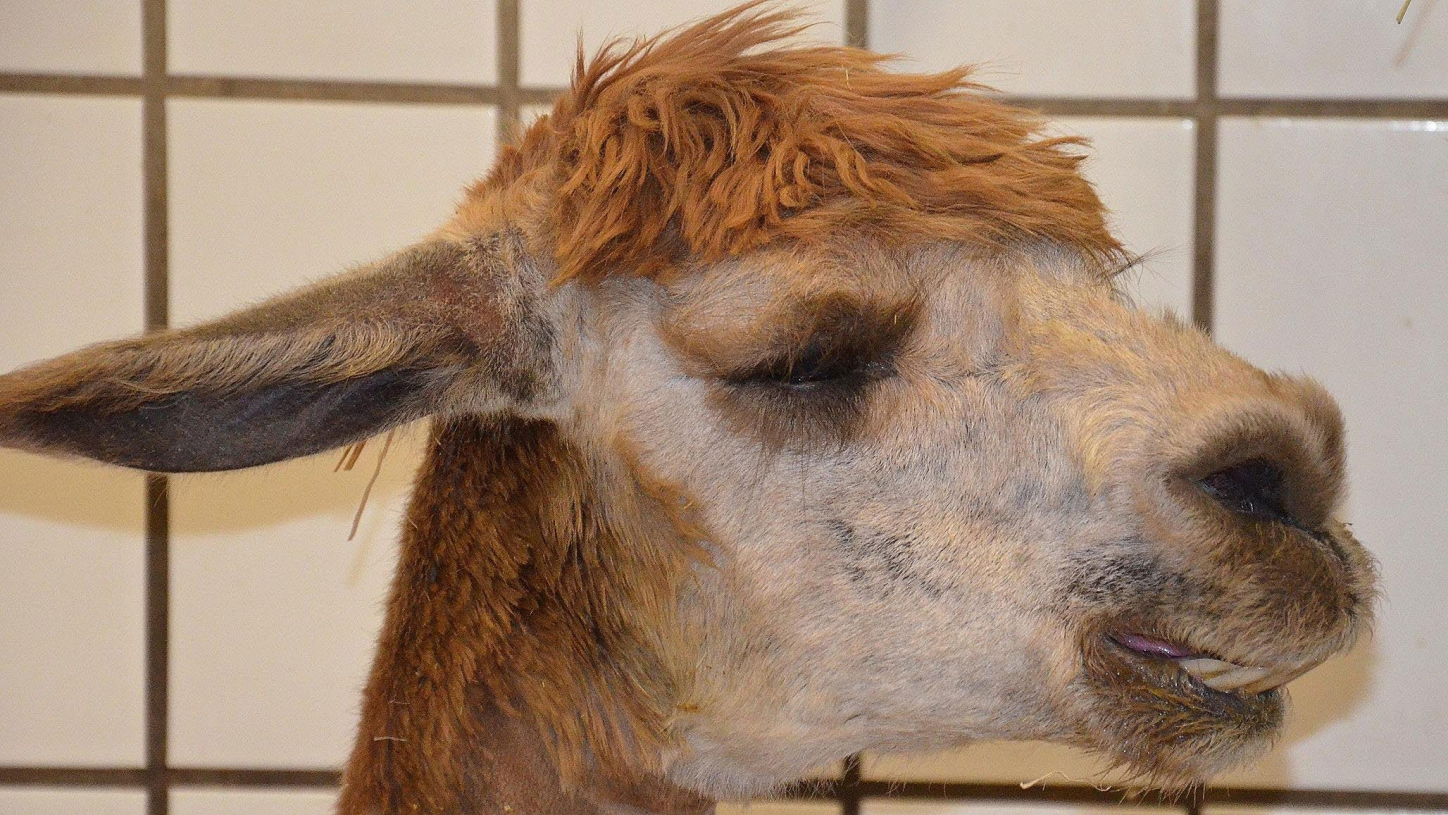
Alpaca 1 showed apathetic behavior, distended nostrils, moaning, and attempted to reguritate unproductively several times. The mouth area was soiled with saliva and food, which indicated previous successful regurgitation

The following clinical signs were observed at the clinic at admission: Alpaca 1 was lying in sternal recumbency in the transport vehicle and was only able to stand up with support. The other animals did not show any abnormal behavior at first sight. The clinical examination on presentation revealed the following findings:

Alpaca 1: The nutritional status of alpaca 1 was estimated according to the body condition score (BCS) system with a score of 3.5 on a scale of 1 to 5 (Wagener et al. 2023). The animal was recumbent in a sternal position, but was able to stand up on its own during the examination. It frequently rolled over its back and showed wheezing and open mouth breathing. The area around the mouth and nose was soiled with food. This female alpaca also retched several times, but no stomach contents were excreted (Fig. 1). Breathing was compressive and abdominally accentuated. Auscultation of the heart and lungs revealed no abnormal findings and auscultation of the first compartment (C1) exposed no clearly demarcated contractions. The conjunctivae of the animal were of physiological color. Examination of episcleral vessels suggested elevated permeability. Moreover, the animal repeatedly moaned during the examination. The body temperature on the following morning was severely decreased (34.1 °C).

Alpaca 2: The BCS of alpaca 2 was estimated to be 3.5. The animal stood in a physiological position, but had a slightly subdued behavior. Auscultation of the heart revealed arrhythmia, but this was not further characterized. The heart rate during auscultation varied between 50 and 80 beats/min (normal range 60–90 beats/min (Niehaus 2022). Auscultation of the lungs showed no abnormal findings, the respiratory type was costoabdominal. The conjunctivae were reddened. Examination of episcleral vessels suggested elevated permeability of episcleral vessels. After a few minutes, the condition of this alpaca deteriorated slightly. Like alpaca 1, it retched several times without regurgitation of stomach contents.

Alpaca 3: The animal did not show any clinical abnormalities. The BCS was rated as 3.5.

Alpaca 4: The animal showed no abnormalities in the clinical general examination except for a slightly soiled mouth area. The respiratory rate was 28 breaths/min and the heart rate 64 beats/min, with intensity, rhythm, and separation corresponding to the norm. The BCS of the animal was 4.

### Initial treatment

Due to the poor general condition of alpaca 1, a venous catheter was inserted in the left jugular vein for drip infusion. However, due to the frequently and uncoordinated rolling of the animal, an infusion was later not performed.

The following drugs were administered to the animals in herd 1 (alpaca 1 in each case via the venous catheter, the other two females in each case subcutaneously): 50 mg/kg metamizole sodium and 0.4 mg/kg N-butylscopolaminium bromide (Buscopan compositum®, Boehringer Ingelheim Vetmedica GmbH, Rohrdorf, Germany), 0.2 mg/kg dexamethasone (4 mg/ml dexamethasone, Bela-pharm GmbH & Co. KG, Vechta, Germany), 3 mg/kg thiamine hydrochloride (Vitamin B1®, Hevert-Arzneimittel GmbH & Co.KG, Nußbaum, Germany).

Alpaca 1 was also treated with 10 mg/kg amoxicillin i.v. (Amoxisel Trockensubstanz 100 mg/mL®, Selectavet Dr. Otto Fischer GmbH, Weyarn, Germany).

Furthermore, all animals in the first herd were initially given 200 mL of medicinal charcoal (containing 24% of charcoal) in liquid form (Carbodote, Ecuphar GmbH, Greifswald, Germany) via a nasogastric tube. However, this treatment had to be stopped in alpaca 1 because safe fixation of the animal was not possible due to the excitations and the animal showed open mouth breathing while the nasogastric tube was inserted.

Based on the anamnesis and the suspected diagnosis of pharyngeal obstruction, a nasogastric tube was inserted into C1 of alpaca 4. To prevent possible aspiration pneumonia, the animal was given 15 mg/kg amoxicillin s.c. (Duphamox LA 150 mg/ml®, Zoetis, Germany). During the examination and treatment, the animal showed no further abnormalities. At the clinic, it immediately started to eat hay, but began to salivate, retch, and show foaming discharge from the mouth later. A venous catheter was inserted into the jugular vein and a blood sample was taken for a blood gas analysis. Furthermore, the alpaca was given 50 mg/kg metamizole sodium and 0.4 mg/kg N-butylscopolaminium bromide i.v., 0.75 mg/kg omeprazole (OMEP 40 mg®, Hexal AG, Holzkirchen, Germany), 100 mL bicarbonate i.v. (sodium bicarbonate 8.4%®, B. Braun Melsungen AG, Melsungen, Germany) and one liter isotonic full electrolyte solution (60 mL/h) i.v. (Sterofundin®, Braun Vet Care GmbH, Tuttlingen, Germany) were administered. Furthermore, the animal was administered 100 mL of the liquid charcoal preparation and additionally 500 mL of water via a nasogastric tube. In addition, the animal received 3 mg/kg thiamine hydrochloride s.c.

## Laboratory findings

Hematological examination of the samples revealed neutrophilia with band neutrophils in alpaca 1. Leucocytosis and neutrophilia were diagnosed in alpaca 2 (Table 1). The differential blood counts of alpaca 3 and alpaca 4 were unremarkable.

**Table 1:** Laboratory findings in the four animals

Parameter	Unit	Reference	Alpaca 1	Alpaca 2	Alpaca 3	Alpaca 4
Hematology						
Leukocytes	G/L	8–16	15.4	20.8	12.1	10.9
Hemoglobin	g/L	110–161	154	139	163	138
Hematocrit	L/L	0.26–0.37	0.36	0.32	0.32	0.32
MCHC	g/L	411–454	428	434	509	431
Lymphocytes	G/L	1.1–5.2	1.85	2.70	2.61	1.69
Segmented neutrophils	G/L	3.4–9.1	12.2	17.2	6.68	8.39
Band neutrophils	G/L	0-0.10	0.62	0.10	0.12	0.33
Eosinophils	G/L	0.8–3.4	0.46	0.21	2.31	0.22
Basophils	G/L	0-0.2	0.0	0.1	0.06	0.0
Monocytes	G/L	0.2–0.9	0.31	0.52	0.36	0.27
NLR		0.5-2.9^a^	6.93	6.41	2.61	5.16
Anisocytosis			+	+	+	+
Poikilocytosis					(+)	+
Polychromasia			(+)	(+)		
Clinical chemistry						
Total bilirubin	µmol/L	0.2–1.01	3.74	n.d.	1.53	n.d.
Total protein	g/L	56.2–70.4	74.6	74.9	62.3	72.8
Albumin	g/L	28.4–37.4	53.4	51.9	41.3	48.8
Globulin/Albumin			0.4	0.44	0.51	0.49
CK	U/L	43–276	535	488	297	31
ASAT	U/L	155–248	96	93	105	101
GLDH	U/L	4-21.2	5	5	5	3
AP	U/L	30–144	49	77	39	149
GGT	U/L	15–43	14	18	23	
Creatinine	µmol/L	104–168	280	256	206	148
Urea	mmol/L	4.5–9.1	9.61	8.03	6.39	5.77
L-Lactate	mmol/L	0.6-4.9^b^ total lactate	n.d.	n.d.	n.d.	6.92
D-Lactate	mmol/L	n.d.	n.d.	n.d.	n.d.	0.05
Calcium	mmol/L	2.1–2.5	2.61	2.61	2.36	2.48
^1^Ca^2+^	mmol/L	1.03-1.31^c^	1.16	1.29	n.d.	1.33
Magnesium	mmol/L	0.8–1.1	1.05	0.96	0.75	0.9
Phosphate	mmol/L	1.1–2.8	0.38	0.59	1.91	0.89
Sodium	mmol/L	148–155	138.1	140.2	145	149.7
Potassium	mmol/L	4.2–5.5	5.35	5.01	4.36	4.29
Trace elements						
Copper (serum)	µmol/L	4.88-10.4^d^	6.8	7.4	8.8	n.d.
Selenium (serum)	µg/L	15.7-206^d^	216.2	212.6	176.1	n.d.
Copper (liver)	mg/kg ww	30-100^e^	58	n.d.	n.d.	n.d.
Selenium (liver)	mg/kg ww	0.25-0.82^e^	0.553	n.d.	n.d.	n.d.
Blood gas						
pH		7.31-7.54^a^	7.26	7.35	n.d.	7,356
BE	mmol/L	- 6.5-8.5^a^	-12	-7.3	n.d.	-8.7
HCO_3_	mmol/L	17.3-32.7^a^	12.9	16.3	n.d.	15.4

Reference values for hematology and clinical chemistry according to Hengrave Burri et al. (2005) or a: according to Hajduk (1992); b: according to Viesselmann et al. (2018) this study only determined total lactate; c: according to Dawson et al. (2011); d: according to Stanitznig et al. (2018); e: according to Puls (1994). Abbreviations: n.d.= not determined, NLR = neutrophil-to-lymphocyte ratio, ww = wet weight.

^1^This value represents ionized calcium in blood and was determined as part of the blood gas analyzis.

The chemical parameters of alpacas 1, 2, and 4 showed hyperproteinemia and hyperalbuminemia. Furthermore, alpacas 1 and 2 showed increased creatine kinase (CK) activities. In addition, elevated plasma creatinine concentrations were determined in the animals on the first farm, with alpaca 1 showing the highest value. Alpacas 1, 2, and 4 also revealed hypophosphataemia. Alpacas 1, 2, and 3 had hyponatremia, which was lowest in alpaca (1). The plasma levels of urea, calcium, and potassium were at the upper reference range in alpaca 1 and alpaca (2). Total bilirubin content in the plasma of alpaca 1 and alpaca 3 was slightly elevated; in alpaca 2, this parameter could not be determined due to technical issues. The other parameters (magnesium, selenium, copper, glutamate dehydrogenase (GLDH), gamma-glutamyltransferase (GGT), aspartate aminotransferase (ASAT), alkaline phosphatase (AP) were unremarkable in these animals.

In alpacas 1, 2, and 4, a blood gas analysis was also performed using a venous blood sample (Wagener et al. 2021), which showed that metabolic acidosis was present in these animals. In addition, L-lactate concentration of 6.92 mmol/L and a D-lactate concentration of 0.05 mmol/L were measured in the plasma of alpaca 4.

### Further clinical process

#### Alpaca 1

The condition of the animal had not improved on the following morning. The alpaca laid in lateral recumbency several times, in between “banging” its head against the ground. However, it was able to stand up on her own, so that in the morning, ruminal fluid (approx. 500 mL) from a donor animal (a cow) and 100 mL Carbodote® were administered via a nasogastric tube. Repeated antibiotic treatment with 15 mg/kg amoxicillin s.c. and repeated administration of a spasmolytic (50 mg/kg metamizole sodium and 0.4 mg/kg N-butylscopolaminium bromide i.v.) were performed. In order to prevent gastric ulcers, 0.5 mg/kg omeprazole was applied i.v. During the day, the condition of alpaca 1 further deteriorated. The animal was mostly recumbent, standing up on its own was only occasionally observed but it swayed when standing and moving. Despite the initiated therapy, the rolling and uncontrolled movements with the head increased so that alpaca 1 was euthanized late that evening due to poor prognosis. The alpaca was euthanized about 30 h after the ingestion of rhododendron leaves as established treatment protocol (Kietzmann 2021) for rhododendron intoxications in small ruminants did not alleviated the clinical signs of the intoxication.

#### Alpaca 2

The condition of this animal improved the following morning. Gagging was no longer observed. Ruminal fluid (approx. 500 mL) and 100 mL Carbodote® were also administered by nasogastric tube. In order to avoid a possible aspiration pneumonia, the alpaca was administered 15 mg/kg amoxicillin s.c. The animal started to eat hay during the following day. Auscultation of the C1 on the second day showed two contractions per minute, auscultation of the heart on the third day revealed arrhythmia. The female was discharged from the clinic that same day on owner’s request.

#### Alpaca 3

The condition of this animal remained constant during the entire stay at the clinic. On the morning of the first day, ruminal fluid (approx. 500 mL) and 100 mL Carbodote® were also administered via a nasogastric tube; no further medication was given due to the good general condition of the animal. This alpaca was also discharged on the third day. Another auscultation of the heart on this day did not reveal any abnormal findings.

#### Alpaca 4

The condition of this animal improved overnight. Auscultation of the heart was performed the next morning and did not reveal any clinical abnormalities. The animal was given 100 mL of Carbodote® using a nasogastric tube. The alpaca was treated intravenously with omeprazole 0.75 mg/kg, dexamethasone 0.3 mg/kg, and 1 L of whole electrolyte solution (60 mL/h). In the course of the same day, the animal started to eat hay. The possibility of ingestion of rhododendron was discussed with the owner, which was confirmed and reported feeding traces on a rhododendron bush. The owner was advised to remove the rhododendron bush from the pasture. On the second day after admission, the animal was again administered amoxicillin 15 mg/kg s.c. As the animal showed no further symptoms, the alpaca was discharged from the clinic.

### Pathological examination of alpaca 1

**Fig. 2:**
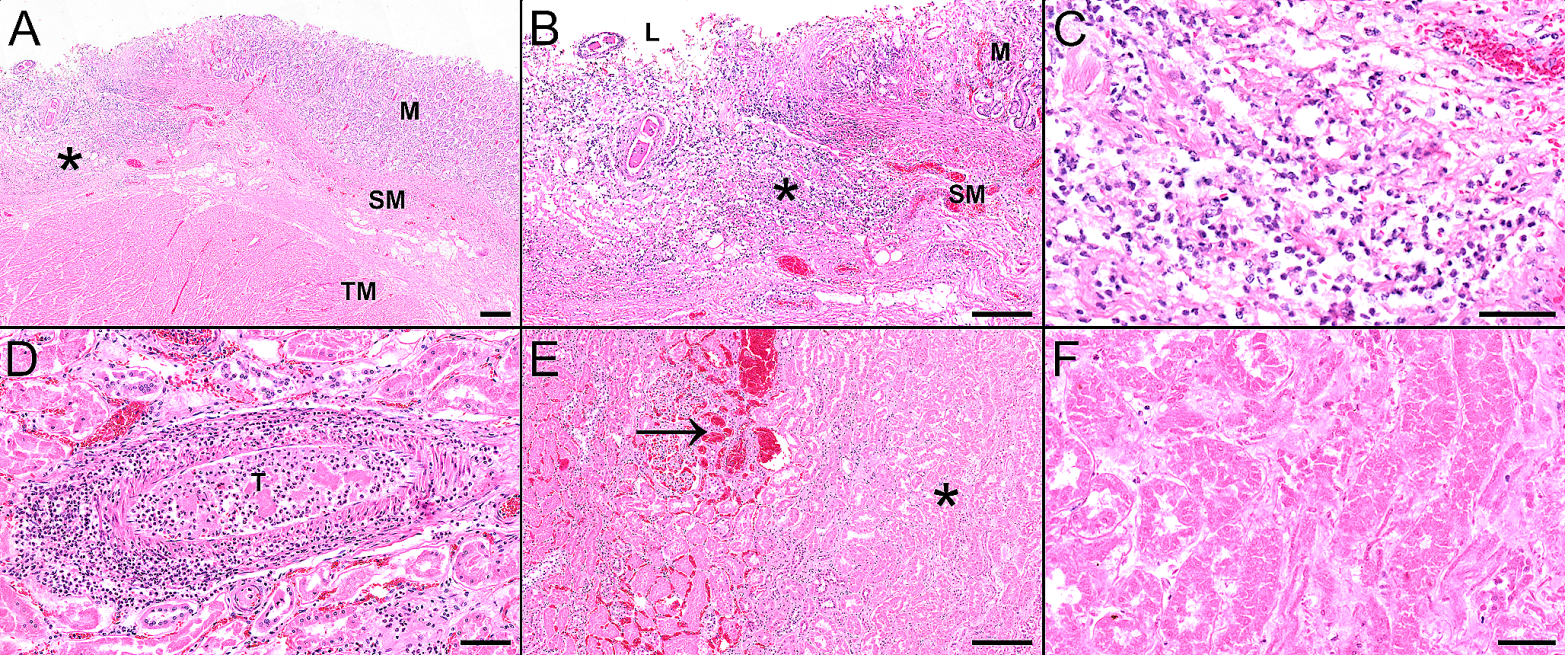
Histopathological findings in the third compartment (A-C) and kidney (D-F) of alpaca 1: (A) The mucosa (M) of the third compartment is extensively ulcerated and displays total destruction (asterisk) reaching into the submucosal layer (SM). TM: Tunica muscularis. (B, C) Higher magnification reveals a marked infiltration of the destructed area with neutrophils and macrophages as well as acute hemorrhages. (D) The thrombosed blood vessel (T) displays a prominent neutrophilic infiltration of the destructed vascular wall as well as the perivascular space. (E) The renal cortex shows an acute infarct with extensive necrosis (asterisk) and adjacent zone of reactive hyperemia (arrow). (F) In a higher magnification, the extensive necrosis of tubular epithelial cells with prominent karyopyknosis and karyolysis can be appreciated. Hematoxylin and eosin stained sections of formalin-fixed and paraffin-embedded tissue. Scale bars: A, B, E: 200 μm; C, D, F: 50 μm

Alpaca 1 was referred to the Department of Pathology, University of Veterinary Medicine Hannover, Germany for a full post-mortem examination. At necropsy, the mucosa of compartment 3 (C3) was diffusely and severely hyperemic and displayed multifocal up to 2 cm in diameter sized ulcerations. The botanical examination of dried forestomach contents revealed a composition of 95% sweet grass (Poaceae) and 5% rhododendron (*Rhododendron* spp.). Small and large intestine contained liquid ingesta. The lung presented with moderate, diffuse, acute hyperemia as well as mild, acute, diffuse, alveolar edema. There were no signs of inflammation and/or aspirated foreign material. The hyperemia and edema were therefore interpreted as agonal changes. The renal cortices of both kidneys showed multifocal pale areas. Within the musculature of the abdominal wall, severe multifocal to coalescing acute hemorrhages were observed. The left uterine horn contained a fetus with a crown-rump length measuring 2 cm. The abdominal cavity was filled with approximately 100 mL serosanguineous effusion. Histopathologically, compartment 2 (C2) and C3 showed a mild to moderate multifocal lymphocytic and plasmacytic inflammation and C3 also acute ulceration already observed macroscopically (Fig. 2A-C). The cortex of one kidney showed a thrombosed blood vessel with an associated mild to moderate purulent perivascular inflammation and vasculitis. Adjacent to the thrombosed blood vessel, an extensive acute renal infarct was visible. Furthermore, the other kidney displayed multifocal mineralization in lumina of renal tubules as well as extensive renal infarcts (Fig. 2D-F). The urea concentration of ocular aqueous humor collected post mortem measured 180 mg/dL (reference range: < 50 mg/dL). The liver showed mild diffuse hepatocellular vacuolization and mild multifocal lymphohistiocytic inflammation with loss of single hepatocytes. The small intestine displayed inflammatory changes characterized by an infiltration with low numbers of lymphocytes and plasma cells with detection of single nematode eggs within the lumen. In adipose tissue located adjacent to the pancreas and adrenal gland, moderate acute hemorrhages were visible. Tracheobronchial and mesenteric lymph nodes showed signs of blood resorption. In addition, a moderate follicular hyperplasia of mesenteric lymph nodes and a mild multifocal lymphohistiocytic and purulent tracheitis was present.

## Discussion

In the four cases described above, the following clinical signs were observed, listed in decreasing frequency: three animals showed increased salivation and retching, bruxism, apathy and decreased C1 motor function. Higher episcleral permeability was suspected in two animals. The majority of symptoms (uncoordinated gait, uncontrolled head movement, open mouth breathing, arrhythmia, and repetitive rolling) were observed only in individual animals. Similar non-specific symptoms have been described previously in alpacas after rhododendron ingestion (Crawford 1999). Even though the symptom retching is not pathognomonic for rhododendron poisoning, the possibility of rhododendron poisoning should be ruled out when retching occurs, as retching is not a frequently observed symptom in alpacas. One of the animals (alpaca 3) did not show any clinical abnormalities leaving an ingestion of rhododendron questionable. A further possibility to secure the diagnosis of rhododendron poisoning is the quantification of grayantoxin in feces (Puschner et al. 2001), ingesta, serum and urine (Puschner 2003). However, these method require specialized laboratories as the different grayanatoxins are quantified by liquid chromatography and mass spectrometry (Puschner 2003).

Hematologically, neutrophilia was observed in alpaca 1, although it is unclear whether these changes are directly related to the ingestion of rhododendron, or are secondary to stress or other diseases (Neubert et al. 2022). The neutrophil-to-lymphocyte ratio (NLR), which was well above the reference value in alpacas 1, 2 and 4, could be an indication of stress. Little is known about the NLR in alpacas, but a stress-related increase in this parameter has been described in other species before (Kim et al. 2005; Swan and Hickman 2014). However, it is also possible that the neutrophilia was a consequence of a local mucosal irritation with consecutive inflammation. The increased activity of creatine kinase and the increased concentration of L-lactate in the plasma indicate a strain in myocytes, which can be explained by the intense uncoordinated movements. Correspondingly, high activities of creatine kinase were described in the plasma of sheep with rhododendron poisoning (Hosie et al. 1986). Puschner also reported increased aspartate aminotransferase and alanine aminotransferase activities in rhododendron poisoning as a consequence of uncontrolled muscle contraction (Puschner 2003). Nonetheless, those chemical findings could not be observed in the animals described here. In addition, increased creatinine concentrations were detected in three animals, combined with increased plasma urea concentration in one animal. Although it is not possible to distinguish between renal and prerenal azotemia on the basis of the present clinical and laboratory diagnostic findings, the bilateral renal infarctions in alpaca 1 suggest a predominantly renal azotemia. The latter has also been described in SACs in connection with acorn or oleander poisoning, which was probably due to the direct nephrotoxic effect of tannic acids and their metabolites (pyrogallol) (Chamorro et al. 2013), or likewise as consequence of acute renal infarctions (Grüss and Priymenko 2009). Postrenal azotemia, which has been observed in urolithiasis (Duesterdieck-Zellmer et al. 2014; Schregel et al. 2022), can be excluded due to undisturbed urine production in all animals. The strong salivation and uncoordinated regurgitation as well as the reduced water intake of the animals probably induced dehydration, followed by electrolyte imbalances and hemoconcentration, indicated also by elevated total protein and albumin concentrations in the plasma (Grosche et al. 2007). Furthermore, metabolic acidosis was observed in the three symptomatic alpacas. Metabolic acidosis can be explained by a loss of bicarbonate due to extreme salivation and impaired secretion of protons in the tubules of the kidneys (Ortiz et al. 1974; Constable et al. 2020). The high concentration of L-lactate is a consequence of anaerobic glycolysis, which may have been triggered by the seizure and the associated increased muscle activity (Fitts 1994). There is no indication that the elevated lactate levels result from a intraluminal production of lactate by the metabolom of the gut, due to the low level of D-lactate. Nevertheless, the increased plasma L-lactate concentrations may also have been caused by hypovolemia due to dehydration (Constable et al. 2020). Hypophosphatemia is frequently found in alpacas and is probably not primarily due to rhododendron poisoning but to an insufficient supply of vitamin D_3_ (Van Saun et al. 1996), but can also be a consequence of impaired tubular function and necrosis of tubular epithelial cells, demonstrated in case 1.

There are several descriptions of rhododendron poisoning in small ruminants and the symptoms described are similar to those of the alpacas presented here (Hosie et al. 1986; Thiemann 1991; Eo and Kwon 2009). At this point it should be emphasized that only alpaca 1 had verifiably ingested rhododendron. The other cases were suspected ingestions of rhododendron, because the non-specific clinical signs such as uncoordinated regurgitation, colic, paresis, anorexia, and convulsions, made it difficult to confirm the diagnosis of rhododendron poisoning. If such an intoxication is suspected, both the evidence of ingestion and the feeding management should be documented immediately. In retrospect, it is difficult to explain the motivation of alpacas to ingest rhododendron. It can be assumed that the highly specialized tongue and cheek papillae of this species enable them to assess the quality of their forage plants either preingestively (by smell) or postingestively (by taste) on the basis of volatile and non-volatile ingredients with a signaling character (Goździewska-Harłajczuk et al. 2015). Rhododendron is not native to the original habitat of alpacas (Popescu and Kopp 2013). For this reason, a co-evolution between alpacas and rhododendron is unlikely. Accidental ingestion could therefore be a possibility. The animals on both farms had been moved only a few months previously and were kept in rotating pasture system. One may speculate whether in the only proven case of alpaca 1, this individual aimed to ingest rhododendron in the context of exploratory behavior. In some published descriptions, rhododendron poisoning in herbivores was fatal (Hough 1997; Crawford 1999; Eo and Kwon 2009), with documented lethal doses for plants containing grayanotoxin in ruminants 0.2–0.6% of body weight (Puschner 2003; Jansen et al. 2012). A duration of 3–14 h between the ingestion of rhododendron and the appearance of symptoms has been reported (Puschner 2003; Jansen et al. 2012). The high variation of lethal doses might originate from the high interspecific variation of Grayanatoxins in leaves of *Rhododendron* spp. (Fattorini et al. 2023). Symptoms are described to subside within one to two days after the end of exposure (Puschner 2003; Jansen et al. 2012). *Rhododendron* spp. contain diterpenes (grayanotoxins), which initially cause local mucosal irritation in the gastrointestinal tract, but can later lead to permanent depolarization of neurons and consecutive neurological signs or cardiac arrhythmia (Puschner 2003). As no electrocardiodiagram-examination was performed, the origin of cardiac arrhythmia was not further classified. However, sinus arrhythmia is frequently diagnosed in alpacas (Ferasin et al. 2005). The diffuse hyperemia of the gastrointestinal tract and the mucosal ulcerations in C3 could be the result of mucosal irritation due to uptake of *Rhododendron* spp. However, gastric ulcerations are frequently caused by stress, NSAIDs, and uremia (Neubert et al. 2022). We assume that treatment of alpaca 1 with metamizole had only little effect on mucosal irritation, as gastroprotective treatment with omeprazole was also given. The inflammatory changes in the gastrointestinal tract probably served as an entry for bacteria and/or endotoxins and could have led to sepsis. Correspondingly, bacteremia and/or endotoxemia might have caused disseminated intravascular coagulopathy with thrombus formation. The acute renal infarcts and hemorrhages might be a consequence of thrombus formation. The gastrointestinal nematode eggs detected in the small intestine are frequent findings and probably of strongylid origin (Neubert et al. 2022). In gastrointestinal tracts of alpacas, Strongylidae of the genera *Haemonchus*, *Cooperia*, *Trichostrongylus*, *Ostertagia*, *Nematodirus*, and *Oesophagostomum* are often found (Franz et al. 2015) and the nematodes sometimes exhibit resistance to anthelmintics (Galvan et al. 2012). Additionally, the histological examination of alpaca 1 revealed follicular hyperplasia of a mesenteric lymph node representing an immunoreaction that can be induced by inflammatory changes in the intestinal tract. The mild lymphohistiocytic and purulent tracheitis was most likely caused by bacterial infectious agents, but probably did not lead to clinically significant impairments.

### Therapy and aftercare

There is different information on the treatment of rhododendron poisoning in ruminants in the literature. Usually, only symptomatic treatment can be given due to the lack of an antidote (Jansen et al. 2012). Mayer recommends rumenotomy to remove the ingested rhododendron, if necessary, antibiotic treatment to prevent possible aspiration pneumonia, and fluid intake to counter hypotension. Depending on the severity of the poisoning symptoms, the increased risk of aspiration pneumonia should also be considered. In goats suffering from intoxication with *Rhododendron* spp., administration of morphine has been described as potential intervention (Mayer 1991). However, this is not an option in the European Union due to the classification of alpacas as food-producing animals. Therefore, a licensed active substance for food-producing animals is lacking (Commission Regulation (EU) No 37/2010). Kietzmann (2021) recommends administering of up to 100 mg i.m. of ephedrine sulfate per animal if the heart rate is reduced (Kietzmann 2021), which is also not permitted for food-producing animals (Commission Regulation (EU) No 37/2010). Some case reports have also suggested a circulatory enhancing effect of caffeine. Black used oxytetracycline, multivitamin preparations, vitamin B12, and oral administration of 50 mL of “warm, strong, sweet coffee” every 15 min for 8 h (Black 1991). In one case, goats poisoned with rhododendron were given paraffin oil and “cold tea” (Humphreys et al. 1983).

For treating rhododendron poisoning in SAC, Lakritz advises oral administration of 10–20 g magnesium oxide and 100–200 g activated charcoal, but warns against administering it to animals showing uncoordinated regurgitation (Lakritz 2022). Additionally, atropine might be administered to treat cardiac arrhythmia. A further treatment option might be an intravenous infusion of a lipid emulsion, as grayanotoxins are lipophilic. Successful interventions in ruminants (Bischoff et al. 2014) and SACs(Dixon et al. 2021) have been described This intervention was not performed, as the regime of lipid emulsion infusion has not been established to the clinic. However, side effects of lipid emulsion infusion are not well-characterized in ruminants (Bischoff et al. 2014).

### Treatment regime

Rumenotomy was not performed because of the highly disturbed circulation and the associated greatly increased risk of anesthesia (Mayer 1991). We administered dexamethason to support the circulation, an infusion with electrolyte solution to compensate for potential dehydration and to maintain circulation as well as oral administration of activated charcoal preparations to bind the toxins in the rumen. Furthermore, imbalances in the acid-base balance should be corrected. We also suggest to administer vitamin B1 (Thiamin) systemically to prevent thiamin deficiency, which might occur as a late consequence of a disturbance of the microbiome in the C1 (Lakritz 2022). If available, a slightly later oral administration of ruminal fluid from a donor animal (in the clinic, ruminal fluid from the rumen fistula of a cow is usually used, but it can also be obtained by gavage) can support the regeneration of the ruminal microflora (Lakritz 2022). If the animals also show uncoordinated regurgitation, antibiotic prophylaxis should be considered if there is a risk of aspiration pneumonia. In addition, hay is offered freely to the animals to encourage them to feed, thus stimulating C1 activity, but feeding of concentrates is avoided.

## Conclusion

Although four alpacas in our case report had been in contact to rhododendron, only one animal showed severe clinical symptoms, which led to the decision to euthanize the animal due to poor prognosis. Rhododendron poisoning can occur in alpacas as well as in small ruminants. The clinical diagnosis is not always clear due to many non-specific clinical signs of varying degrees. When animals are presented with salivation, retching, or uncoordinated regurgitation, apathy, dehydration, and reduced forestomach motility, rhododendron ingestion should be taken into consideration. Possible access to rhododendron should always be asked in the anamnesis of animals showing previously mentioned symptoms. Rhododendron poisoning in alpacas, as in small ruminants is an emergency with an uncertain outcome. Treatment should be symptomatic with rehydration and administration of activated charcoal. In addition, secondary diseases such as kidney damage or aspiration pneumonia should be considered.

## Data Availability

The original contributions presented in the study are included in the article/supplementary material; further inquiries can be directed to the corresponding author.

## Abbreviations

BW: Bodyweight
SAC: South American camelids
